# A New Preparation of Cotton for Stanching Hemorrhage

**Published:** 1871-01

**Authors:** 


### A NEW PREPARATION OF COTTON FOR STANCHING HEMORRHAGE.

Dr. Ehrle, of Isny, calls attention, in the *Schwabischer Merkur*, to a simple preparation of cotton, which he has found of great service in surgical operations followed by great effusion of blood. The mode of preparation is as follows: American cotton of the best quality should be cleansed by boiling it for an hour in a weak solution of soda (about 4 per cent.), then repeatedly washed in cold water, and dried. By this process it will be perfectly disinfected and adapted to more ready absorption. After this it should be steeped once or twice, according to the degree of strength required, in liquid chloride of iron, diluted with one-third water, pressed, and thoroughly dried in the air,—*neither in the sun nor by the fire*,—then lightly pulled out. The cotton so prepared will be of a yellowish-brown color It must be kept very dry, as it is affected by the damp. Lint may be similarly treated, but the fine texture of the cotton renders it preferable. When placed on a fresh wound, it causes a moderate contraction of the tissue, and gradually coagulates the blood in and beyond the injured veins, thus closing the source of the effusion. This property of the chloride of iron is increased by the dryness of the cotton and the extended surface offered for the development of the chemical action.—*Pharm. Journal* (*London*).

---

Dr. E. Sommer, in *Zeitschrift fur Rationelle Medicin*, declares that sleep is the result of a *deoxygenation* of the organism. "The blood and the tissues possess the property of storing up the oxygen inhaled, and then supplying it in proportion to the requirements of the economy. When this state of oxygen is exhausted, or even becomes too small, it no longer suffices to sustain the vital activity of the organs, the brain, nervous system, muscles, &c., and the body falls into that particular state which we call sleep. During the continuation of this deep repose, fresh quantities of oxygen are being stored up in the blood, to act as a supply to the awakened vital powers. Rest produces though in a less degree, the same effect as sleep in reducing the expenditure of oxygen.—*National Med. Journal.*

